# Optimization of Bioactive Compound Extraction from Iranian Brown Macroalgae *Nizimuddinia zanardini* with Ultrasound and Microwave Methods Using Fuzzy Logic

**DOI:** 10.3390/foods13233837

**Published:** 2024-11-28

**Authors:** Atefe Taherkhani, Akram Sharifi, Mohamed Koubaa

**Affiliations:** 1 Department of Food Science and Technology, Qazvin Branch, Islamic Azad University, Qazvin 82CM+Q6X, Iran; atefe.taherkhani13@gmail.com; 2 Université de Technologie de Compiègne, ESCOM, TIMR (Integrated Transformations of Renewable Matter), Centre de Recherche Royallieu-CS 60319, 60203 Compiègne CEDEX, France

**Keywords:** *Nizimuddinia zanardini*, fuzzy logic ranking, antioxidant activity, bioactive compound, novel extraction methods

## Abstract

In this study, three extraction methods of bioactive compounds from the brown algae *Nizimuddinia zanardini* were ranked using the fuzzy weighting system in two stages, ranking between different conditions and choosing the optimal conditions for each extraction method separately. The inputs included extraction yield (EY), antioxidant activity (DPPH), total flavonoid content (TFC), total phenolic content (TPC), total phlorotannin content (TPhC), time, temperature, power, and cost. The top ranks of the first phase output included: Maceration Extraction (ME) with a score of 52.67, Ultrasound-Assisted Extraction (UAE) with a score of 54.31, and Microwave-Assisted Extraction (MAE) with a score of 73.09. The results of the second stage indicated that the lowest and highest extraction yields were obtained using UAE and MAE, respectively. The TFC in the UAE was determined as 103.29 mg QE (Quercetin Equivalent)/g as the lowest value and, in the ME, 140.95 mg QE/g was the highest value. The highest and lowest TPC and TPhC were observed with MAE and UAE, respectively. DPPH decreased in UAE, MAE, and ME, respectively. According to the fuzzy weighted results and considering the purpose of extraction, MAE can be introduced as the optimal method for extracting bioactive compounds from *N. zanardini*. The findings on extraction methods underscore the potential to reduce costs and improve the yields of bioactive compounds, such as antioxidants and polyphenols, thereby enhancing the economic viability of functional foods and nutraceuticals.

## 1. Introduction

In Asia and lately worldwide, seaweeds represent a significant part of the human diet due to their high concentration of polysaccharides, vitamins, minerals, and polyunsaturated fatty acids (PUFA). As a result of the nutritional value of seaweeds and their use in raw food products, as well as their bioactive molecules, such as carotenoids, phlorotannins, fucoidan, fucoxanthin, and phloroglucinol, consumers are increasingly choosing foods that contain seaweeds as healthy alternatives [1]. Marine algae, also known as seaweeds, can be divided into three classes depending on their pigment composition and chemical structure. These three types of algae are green algae (Chlorophyta), brown algae (Phaeophyta), and red algae (Rhodophyta) [2]. *Nizimuddinia zanardini* is a brown seaweed of the Sargassaceae family. The *Nizimuddinia* genus is known only from this species. It grows in the Persian Gulf (Gheshm Islands and Chabahar, Iran), the Arabian Sea coasts (Yemen, Oman, and Pakistan), and southwest Asia [3]. Due to its high density and biomass along Iran’s east coast, this species was selected for the present study [4]. In addition, the fucoxanthin content, lipid content, and antioxidant properties of *N. zanardini* have been reported [4]. Similarly, another study evaluated the cytotoxic properties of hydroperoxy sterol extracted from this algae [5]. The bioactive compounds found in *N. zanardini*, particularly phlorotannins and fucoxanthin, offer valuable health benefits that can be effectively utilized in the food industry [3]. Their antioxidant, anti-inflammatory, and anti-obesity properties position them as ideal candidates for functional foods targeting metabolic health, weight management, oxidative stress reduction, and chronic disease prevention. As consumer demand for health-promoting ingredients continues to rise, integrating these bioactive compounds into food products offers a significant opportunity for innovation in the functional foods sector [6,7]. Despite these benefits, no studies have been conducted on the novel extraction and characterization of *N. zanardini* crude extract and its potential bioactivities.

Adopting novel extraction methods, such as UAE and MAE, offers significant benefits for the food industry by reducing production costs and environmental impact. These methods enhance the efficiency of bioactive compound recovery, thereby lowering material and energy costs while simultaneously contributing to a more sustainable and eco-friendly production process. As the food industry increasingly embraces green technologies, these extraction techniques are poised to play a pivotal role in the future development of functional foods [8].

The recovery of bioactive compounds from plants is accomplished in five distinct stages. This methodology consists of (1) macroscopic pretreatment, (2) separation of macro- and micro-molecules, (3) extraction, (4) purification, and (5) product preparation. The extraction is one of the most critical steps [9]. In recent years, non-conventional extraction methods have become increasingly popular in academia and industry due to their low organic chemical requirements, rapid extraction processes, and high yields [10,11]. To increase the selectivity and yield of bioactive compounds, extrusion, accelerated solvents, ohmic heating, supercritical fluids, pulsed electric fields, ultrasound, microwave heating, and enzyme digestion have been investigated as non-conventional technologies [12,13].

Compared to conventional extraction methods such as maceration or shaking, ultrasound and microwave extraction are considered green extraction methods since they decrease extraction time, increase yield, and reduce solvent consumption [14,15].

MAE of bioactive compounds from four seaweed species (*Ascophyllum nodosum*, *Laminaria japonica*, *Lessonia trabeculate*, and *Lessonia nigrecens*) [16], UAE of *S. wightii*, *G. edulis*, *U. rigida* [17], ME of *Dictyota dichotoma* and *Padina pavonica* [18], and UAE, MAE, and ME for *A. nodosum* and *Padina pavonica* [14,15] are some of the research studies that have been conducted in this field.

Each of these methods enhances the extraction of bioactive compounds. However, their ranking is influenced by numerous factors, including temperature, time, antioxidant activity, yield, cost, selectivity, total bioactive compounds, etc., all of which can be challenging to determine. Decision Support Systems (DSSs) effectively manage decision-making processes [19]. Hence, fuzzy logic systems facilitate the exchange of concepts between mathematics and humans, as well as the formalization of linguistic expressions [20,21,22,23]. Fuzzy logic models have successfully been applied to numerous food processing and technology studies to analyze information and improve decisions [10,11,24]. Consequently, this study aims to develop an efficient fuzzy system of bioactive compounds depending on a variety of input data to rank different conditions and extraction techniques of bioactive compounds from *N. zanardini*.

## 2. Materials and Methods

### 2.1. Macroalgal Biomass and Processing

*N. zanardini* (Schiffner) P.C. Silva was harvested in January 2022 from the Oman Sea in the south of Iran (Chabahar Bay: 25°20′53″ N and 60°28′1″ E). The Off-Shore Fisheries Research Center determined the taxonomic group of the seaweeds. After harvesting, it was thoroughly rinsed with fresh water, sun-dried, ground, and sieved to a uniform particle size (≈1 mm), vacuum-packed, and stored in a cool and dry place for further experimentation. All chemicals, reagents, and solvents used were of adequate analytical grade and were obtained from Merck KGaA (Darmstadt, Germany) and Sigma-Aldrich (St. Louis, MO, USA).

### 2.2. Extraction Procedure

According to the preliminary test (yield of extraction), the sample-to-solvent ratio was set at 1:10, and 50% ethanol was used to extract the sample. The selection of 50% ethanol as an extraction solvent in this study was based upon previous findings where 50% ethanolic solution resulted in the highest recovery of total phenols from macroalgae compared to 30% and 70% ethanol solutions [25]. Before extracting the samples, the mixtures were stirred for 30 min at room temperature and 200 rpm agitation speed. The following extraction steps were performed: in Maceration Extraction (ME), the mixture was extracted at different temperatures (30, 45, and 60 °C) in the dark for 12 and 24 h during agitation at 400 rpm. Ultrasound-Assisted Extraction (UAE) was performed using an ultrasonic bath (Transsonic 100H, Elma Schmidbauer GmbH, Singen, Germany) at 37 kHz and a power of 100 W. Extractions were performed at various temperatures (30, 45, and 60 °C) for 1 and 2 h. Microwave-Assisted Extraction (MAE) was carried out using an MAE instrument (Microwave-Oven, ME341, Multiwave-3000), built, set up, and used in the laboratory of the Iranian Research Organization for Science and Technology (IROST, Tehran, Iran). The mixture was extracted at different power values (100 and 200 W) for 10, 15, and 20 min. Once the extraction was completed, the samples were placed in an ice bath for 5 min to rapidly decrease the temperature and avoid degradation of the compounds. Afterward, each extract was filtered using Whatman filter paper (No. 1). Optimizing extraction parameters—temperature, time, and power—is critical for maximizing bioactive compound yield in food applications. Temperature enhances extraction efficiency by increasing solubility and diffusion rates, though excessive heat can degrade sensitive compounds like antioxidants and vitamins [26]. Similarly, extraction time affects yield and efficiency; longer durations can improve yield but may reduce bioactivity and increase energy costs. Balancing time is key for quality and cost-efficiency. Power, particularly in MAE, accelerates extraction but risks overheating sensitive compounds, making precise power control essential [27]. Industrial optimization of these parameters improves extraction efficiency, reduces energy and solvent use, lowers costs, and supports sustainable functional food production by preserving bioactive properties and minimizing waste [28].

Therefore, in the current study, the extract was freeze-dried at low pressure and temperature (0.0002 bar and −60 °C, respectively) (Dena Vacuum System, Tehran, Iran). The extracts were then vacuum-packed and stored at −20 °C for further analyses. Based on the solid weight of the extracts, a yield was determined according to Equation (1).

EY (%) = [(solid in extract (g)/raw material (g)] × 100(1)

### 2.3. Bioactive Compounds Analyses

A stock concentration of 1 mg/mL was obtained by dissolving each freeze-dried extract in ethanol. All the phytochemical analyses of the extracts were performed in triplicate (*n* = 3).

#### 2.3.1. Total Phlorotannin Content (TPhC) and Total Phenolic Content (TPC) Analyses

TPhC and TPC were measured using the Folin-Ciocalteu (FC) reagent method, as previously described [25]. A volume of 100 µL of either a sample or a standard was mixed with 2 mL of sodium carbonate solution (2% *w*/*v*). Afterward, 100 µL of FC reagent was added to each mixture, and the reaction was conducted by incubating all the tubes for 30 min at room temperature in the dark. A spectrophotometer (Lambda 35 UV/VIS spectrometer, Perkin Elmer, Waltham, MA, USA) was used to read the absorbance values at 765 nm. Distilled water (free of extract or a standard) containing the other reagents was used as a blank. Gallic acid (>97.5% purity) and phloroglucinol (>99% purity) at concentrations of 100–1500 mg/L were used as standards for TPC and TPhC, respectively. TPC results are expressed as mg GAE (Gallic Acid Equivalent)/g and TPhC as mg PGE (Phloroglucinol Equivalent)/g.

#### 2.3.2. Total Flavonoid Content (TFC)

An aluminum chloride colorimetric method was used to determine the TFC of *N. zanardini* extracts, as previously described [15]. A volume of 250 µL algae extract was mixed with 750 µL ethanol (96%), 50 µL aluminum chloride (10%, *w*/*v*), 50 µL sodium acetate (1 M), and 1400 µL distilled water. After 30 min incubation at room temperature, the absorbance was measured at 415 nm using a spectrophotometer (Lambda 35 UV/VIS spectrometer, USA). The standard calibration curve was plotted using quercetin (1–1100 mg/L). The TFC results were expressed in mg QE (Quercetin equivalent)/g.

#### 2.3.3. DPPH Radical-Scavenging Activity

DPPH (1,1-Diphenyl-2-Picryl-Hydrazil) radical-scavenging activity was performed as previously described [10]. In total, 700 µL of methanolic DPPH solution (100 µM) was added to the 700 µL sample, and the mixtures were incubated at room temperature in the dark for 20 min. The absorbance of the reaction was determined compared with a blank (methanol without DPPH solution) at a wavelength of 515 nm in a spectrophotometer (Lambda 35 UV/VIS spectrometer, USA). The DPPH radical-scavenging activity was calculated according to Equation (2):

DPPH (%) = ((A blank − A sample)/A blank) × 100(2)

### 2.4. Fuzzy Ranking System

A fuzzy ranking system is particularly well-suited for determining the most effective extraction methods in the food industry due to its ability to address the uncertainty, variability, and complexity inherent in real-world food processing scenarios. Fuzzy logic enables food processors to make more robust decisions by incorporating variations in input parameters (e.g., temperature, time, solvent choice), raw material quality, and environmental conditions. These factors can significantly influence extraction efficiency and product quality, rendering traditional deterministic approaches less effective in ensuring consistent outcomes [29]. A fuzzy ranking system was implemented using a previously developed methodology [10]. For this study, 25 investigators were chosen, including professors, researchers, and lecturers who contributed to at least two original studies on the novel extraction methods. Due to the consequence of a data-driven fuzzy methodology, the total fuzzy score of each extraction technique was calculated accordingly: (1) input data fuzzification (antioxidant activity, extraction yield, total phlorotannin content, total phenolic content, total flavonoid content, temperature, time, power, and cost) in a triangle membership function using Equation (3); (2) importance score of each parameter fuzzification based on the related score sheet and Equation (1); (3) the total fuzzy score of each method estimation based on Equation (4); (4) converting fuzzy outputs to crisp numbers (defuzzification) to assist with decision-making; and (5) ranking the extraction methods. The steps described above were implemented in MATLAB 2016b (Mathworks Inc., Natick, MA, USA).

(3) $$s_{r}p=\frac{n_{1}\left(0 0 25\right)+n_{2}\left(0 25 50\right)+n_{3}\left(25 50 75\right)+n_{4}\left(50 75 100\right)+n_{5}\left(75 100 100\right)}{n_{1}+n_{2}+n_{3}+n_{4}+n_{5}}$$

where $s_{r}p$: fuzzy score based on a particular variable (for instance, antioxidant activity, total phlorotannin content, temperature, cost, etc.); $n_{1}$ + $n_{2}$ + $n_{3}$ + $n_{4}$ + $n_{5}$: the total number of experts; $n_{1}$, $n_{2}$, $n_{3}$, $n_{4}$, $n_{5}$: the number of experts giving “very poor/very unimportant (0, 0, 25)”, “poor/unimportant, (0, 25, 50)”, “fair/somewhat important (25, 50, 75)”, “Good/important (50, 75, 100)” and “Very Good/highly important (75, 100, 100)” to each parameter, respectively.

(4) $$S_{r}O=\frac{Q{AA}_{r}+Q{PC}_{r}+Q{PhC}_{r}+Q{FC}_{r}+QY_{r}+Q{Ti}_{r}+Q{Tem}_{r}+Q{Pow}_{r}+Q{CO}_{r}}{\sum Q}$$

where $S_{r}O$: overall score of the extraction method; AA: antioxidant activity based on DPPH; PC: content of total phenolic compounds; PhC: total phlorotannin content; FC: total flavonoid content; Y: efficiency; Ti: time; Tem: temperature; Pow: Power; CO: cost of extraction method; and Q: the importance coefficient of each component.

### 2.5. Statistical Analyses

A fuzzy logic analysis was used to rank each sample, and an analysis of variance (ANOVA) at three replications was performed on the bioactive compounds data. Tukey’s multiple range test was used to analyze the significance of differences between the averages (*p <* 0.05). These steps were implemented in the SPSS (version 25.0) program (IBM SPSS Statistics, SPSS, Inc., Chicago, IL, USA). A correlation relationship was investigated between the antioxidant activities of the extracts and their composition in R software version 4.2.2 (R Core Team, Vienna, Austria). The statistical packages “corrplot” and “ggplot2” were used to create the correlation matrix and “cor.mtest” calculated the *p*-values for Pearson’s correlation matrix.

## 3. Results and Discussion

### 3.1. Effect of Extraction Methods on the Bioactive Compounds and Yields of Extracts

Table 1 presents the results of each extraction technology as mg dried extract/g macroalgae. In general, in the comparison of the extraction methods, the yields of compounds extracted were higher with MAE (yields ranging from 33.19 to 54.04%) compared to other extraction methods (from 17.76 to 28.27 mg dried extract/g macroalgae and from 23.48 to 27.67 mg dried extract/g macroalgae in UAE and ME, respectively). Overall, comparing extraction methods, the yields of compounds extracted from macroalgae were the highest using MAE (at 300 w for 20 min), followed by ME (at 60 °C for 12 h). UAE achieved the lowest yields at 30 °C for 60 min. This could be due to the relatively low output power of the ultrasound bath [30]. The results of another study also indicated that an ultrasound bath yields a much lower yield than conventional or Microwave-Assisted Extraction techniques [31].

The influence of various extraction methods, extraction time, temperature, and power on the extraction of bioactive compounds (TPC, TPhC, and TFC) of *N. zanardini* is shown in Table 1. Regarding technological conditions, temperature, power, and extraction time, there was a significant difference in bioactive compound extraction. As previously demonstrated [32], the TPC, total sugar content (TSC), and other bioactive compounds produced by the brown macroalgae, *Laminaria hyperborean*, *Ascophyllum nodosum*, and *Laminaria digitalta,* harvested in Ireland every season for up to two years, differ substantially. As a result, the concentrations of TPC, TPhC, TFC, total tannin content (TTC), and TSC extracted in this study may be strongly affected by the initial concentration of these compounds in the biomass. Additionally, in another work, the authors found that macroalgal species with strong cell walls containing varying types and amounts of polysaccharides were one of the main obstacles to efficient extraction [33].

As a result of focusing on a unique method, MAE obtained the highest extraction yields of TPC and TPhC from *N. zanardini*. The highest yield of TPC and TPhC were found in extracts produced using MAE from *N. zanardini* (from 410.26 to 897.22 mg Gallic Acid Equivalent (GAE)/g dried extract), respectively. MAE has been successfully applied to brown macroalgae and led to obtaining high polyphenol yields and antioxidant activities; however, these latter may vary depending on the MAE technology used, the extraction conditions, and the biomass (species, season, and collection place) [14]. In another work, the authors obtained low yields of TPhC (9.8 ± 1.8 mg PGE/g extract) from *F. vesiculosus* using optimal MAE parameters (5 min, 75 °C, and 57% ethanol), compared to those obtained using conventional solvent extraction (11.1 ± 1.3 mg PGE/g extract) [34]. Despite this, it was demonstrated that high yields of TPC were obtained using MAE on brown macroalgae (*A. nodosum*, *Laminaria japonica*, *Lessonia trabeculate*, and *Lessonia nigrecens*) irradiated with microwaves (2.45 GHz) at 110 °C for 15 min (5 min climbing and 10 min holding) [16].

Based on the results of the current study, UAE had a significantly lower TPC value than the MAE and ME methods. UAE did not demonstrate any advantage over MAE in this study. In another work, the authors optimized UAE for extracting carbohydrates and polyphenols of the brown macroalgae *Silvetia compressa* [35]. TPhC was reported to be high (10.82 mg PGE/g), with maximum ultrasound power and medium polarity solvents (62.5% ethanol solution). In addition, it was demonstrated that TPC, TPhC, and TFC were effectively extracted from *F. vesiculosus* using UAE for 30 min and 50% ethanol as an extraction solvent [36]. It was also shown that UAE (60 min at 60 °C with 60% ethanol as an extraction solvent) was employed to extract TPC (9.07 mg GAE/g), DPPH (16.11 mg TE/g), and FRAP (9.03 mg TE/g) from *Padina australis* [37]. These studies show that macroalgal cell walls contain different amounts and types of complex polysaccharides based on the species of macroalgae. This poses one of the most significant challenges to efficiently extracting compounds from macroalgae [33]. Furthermore, the amount of bioactive compound in macroalgae differs according to geographical location and harvest time [14]. It is essential to develop a specific extraction strategy for each algal species to achieve the highest yield of bioactive compounds [32].

High levels of TFC (94.29 to 242.67 mg QE/g) were extracted using ME compared to those obtained using UAE and MAE. Similar to these results, the application of ME, except in TFC, did not significantly improve the extraction of bioactive compounds from *N. zanardini*.

Overall, MAE extracted significantly more bioactive compounds from *N. zanardini* than other methods, except for TFC. The integration of bioactive compounds derived from MAE, TPC, and TPhC offers the potential to significantly enhance the health benefits, functional properties, and marketability of food products. Additionally, this approach contributes to sustainability and cost reduction, aligning with the growing demand for health-oriented and environmentally sustainable food solutions [38]. MAE is an effective method for enhancing the yield of bioactive compounds such as total phenolic content (TPC) and total phlorotannin content (TPhC), while also preserving their bioactive properties. This makes MAE particularly suitable for applications in food fortification and health supplements. By ensuring higher concentrations of bioactive compounds without compromising their effectiveness, MAE facilitates the development of functional foods and nutraceuticals that meet the growing consumer demand for health-promoting products [27,39]. It was reported that maximum TPC, TFC, and TSC recovery was obtained when using 75.23% ethanol at 51 °C and a solid/liquid ratio of 1:50 for 73.02 min of extraction of *Asparagus officinalis* root cultivars [40]. In another work, *P. pavonica* extracts with the highest yields of TPC were obtained by MAE (5 min, 60 °C, and 200 W) using 50% ethanol [41]. It was also shown that MAE (3 min, 160 °C, and a water/biomass solvent ratio of 1:30) increased the yield of polyphenols extracted from brown macroalgae (*Carpophyllum flexuosum*) by 70% compared with solid-liquid extraction [41]. A higher temperature resulted in a higher TPC and a 24 times shorter extraction time. This may be caused by the increased interaction between molecules in the solvent at a higher temperature, which increases the compound’s solubility. The results of some studies indicate that high temperatures (>60 °C) produce a higher yield of TPC from brown algae [16,42,43]. Despite the increase in total phenolic content (TPC) due to the hydrolysis of complex phlorotannins into simpler compounds, the high-temperature treatment raises concerns about the thermal susceptibility of phenolic compounds [15].

### 3.2. Effect of Extraction Methods on the Antioxidant Activity

**Figure 1:**
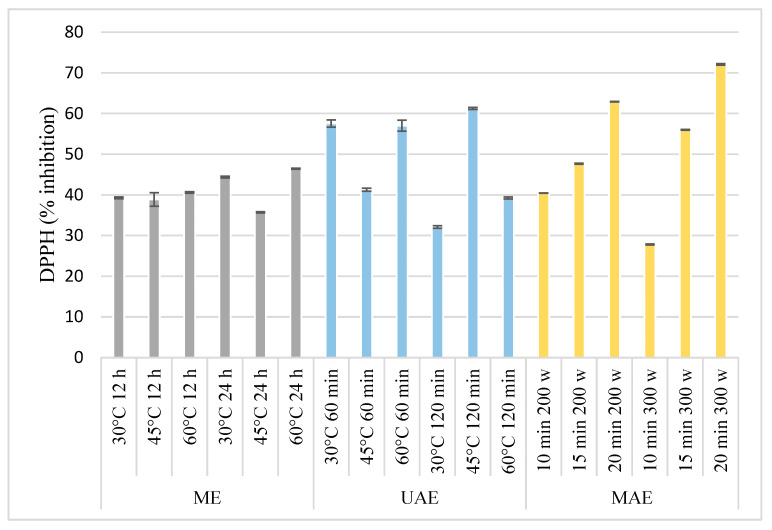
The effect of extraction methods on the DPPH radical-scavenging activity of *N. zanardini* extract. ME: Maceration Extraction, MAE: Microwave-Assisted Extraction, UAE: Ultrasound-Assisted Extraction.

The antioxidant activities (DPPH scavenging activity) of the extracts obtained with the different extraction methods from *N. zanardini* are shown in Figure 1. The antioxidant activities of the extracts varied significantly based on the temperature, technology, and extraction time used. In general, extracts from MAE at 300 w for 20 min had higher DPPH activities than others, and the lowest values were observed in MAE extracts (at 300 w for 10 min).

Regarding free antioxidant activity against DPPH radicals, *N. zanardini* extracts ranged from 27.82 ± 0.13% to 72.09 ± 0.16%. UAE and MAE, both novel extraction methods, achieved a high DPPH scavenging activity. Extraction methods like MAE and UAE enhance food preservation by leveraging high antioxidant activity to prevent oxidative spoilage, thus maintaining flavor, color, and nutritional value in perishable foods [44,45]. One study assessed solvent effects on the yield, phenolic content, antioxidant, and antimicrobial activity in nine brown macroalgae species, showing ethanolic extracts produced the highest yields and phenolic content, with *Undaria pinnatifida* having the greatest values [46]. Another study optimized extracts from three brown seaweeds as natural angiotensin I-converting enzyme (ACE) inhibitors, finding that the α-amylase extraction of *L. nigrescens* had the highest yield and ACE inhibition, outperforming maceration [47]. Comparatively, MAE was the most effective method for extracting compounds from soursop leaves, yielding 33.98% and requiring a shorter processing time than maceration or Soxhlet extraction [48]. MAE reduces solvent and energy usage, offering a cost-effective and efficient process, while preserving bioactive compound integrity, crucial for functional food applications [49].

**Figure 2:**
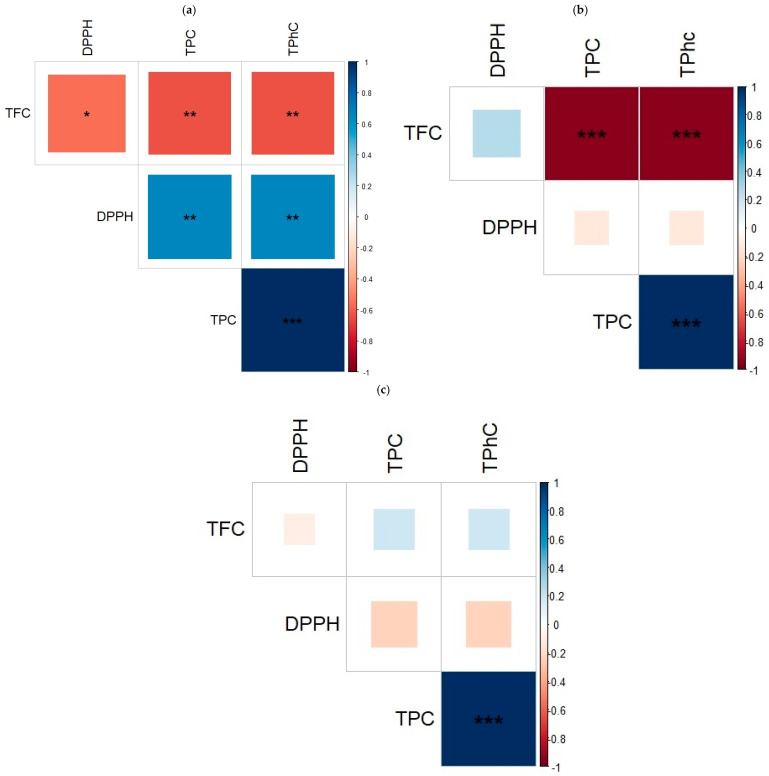
Correlation matrix between the compound and antioxidant activity (DPPH) of extracts from (**a**) ME, (**b**) UAE, and (**c**) MAE. Correlation signs are color-coded (red = − and blue = +) and correlation strengths (0–1) are determined by the depth of each color and the size of the squares. The figure is abbreviated as follows: total phlorotannin content (TPhC), total phenolic content (TPC), 1,1-diphenyl-2-picryl-hydrazil radical scavenging activity (DPPH), and total flavonoid content (TFC). Three statistically significant correlations are presented: * *p <* 0.05, ** *p <* 0.01, *** *p <* 0.001.

According to Figure 2, correlation matrices were examined to investigate the relationship between bioactive compounds and the antioxidant activity of *N. zanardini* extracts. The ME showed a significant correlation between antioxidant activity (DPPH), TPC, TPhC, and TFC. As a result of the UAE study, TPC was positively correlated with TPhC and with TFC. Additionally, TPC and TPhC were positively correlated in MAE. The results of this study are consistent with prior studies that have linked high polyphenol content to high antioxidant capacity in macroalgae [14,50]. As a consequence, macroalgae produce antioxidant bioactive compounds in response to environmental stress to prevent structural and metabolic changes caused by oxidizing agents [51]. Numerous studies have shown that high levels of TPC production and antioxidant activity in macroalgae defend against oxidative stress during the spring and summer seasons [50,51,52]. Therefore, the environment and harvesting season strongly influence macroalgae’s bioactive compounds and antioxidant activity. In addition, studies analyzing TPC, TPhC, and TFC concentrate on their antioxidant properties. This is crucial for the prospective use of polyphenols in high-value applications such as pharmaceuticals, nutraceuticals, and cosmeceuticals [14,15,25].

### 3.3. Fuzzy Ranking

#### 3.3.1. Maceration Extraction (ME) Method

According to the previous sections, optimal conditions varied depending on time and temperature. Thus, it is difficult to determine appropriate conditions for the extraction of bioactive compounds.

Based on previous knowledge, expert systems can be used to solve complex problems [53,54]. This section presents a data-driven fuzzy system that ranks different ME conditions for *N. zanardini* according to the extraction yield, DPPH, TPC, TPhC, TFC, extraction time, and extraction temperature.

The panelists’ responses are listed in Table 2. This table shows that the experts recommended ME at 30 °C for 24 h for TPC, TPhC, and temperature, and ME at 60 °C for 24 h for DPPH and extraction yield. Generally, ME at 60 °C for 24 h scored “good” regarding TPC, TPhC, and temperature. This method also achieved a “fair” score (>50) for the TFC parameter. Although ME at 60 °C for 24 h had a fair to good score in terms of TPC and TPhC, it displayed “very poor” and “poor” performance in terms of TFC and time, respectively.

The importance of each parameter is shown in Table 6. Based on the results of the defuzzification, all extraction factors ranged from important to very important (defuzzification score > 75). It could be concluded from this that the parameters selected for ranking the different condition systems were significant and accurate. High temperatures negatively affect bioactive compounds such as phenolic compounds and phlorotannins, as well as the antioxidant activity of the extract. To extract bioactive compounds from *N. zanardini*, temperature was considered the most important factor.

As shown in Table 7, the fuzzy score for each extraction method was calculated using the combined results of Table 2 and Table 6. The ME at 30 °C for 24 h showed good performance at ambient temperature, causing a fair total score for this method. In total, ME at 30 °C for 24 h achieved a fair (52) score and is suggested for the extraction of bioactive compounds from *N. zanardini*.

#### 3.3.2. Ultrasound-Assisted Extraction (UAE)

This section presents a data-driven fuzzy system that ranks different UAE conditions for *N. zanardini* according to the same variables.

As shown in Table 3, the experts recommended 45 °C for 1 h for TPC, TPhC, and time; UAE at 45 °C for 2 h for DPPH; and UAE at 60 °C for 2 h for TFC and extraction yield as the most suitable extraction conditions. Overall, the UAE scored above “good” for TPC, TPhC, and time at 45 °C for 1 h. This method also achieved a “very poor” score (>50) for the TFC and DPPH parameters.

Table 7 provides the total fuzzy score for each condition. According to this method, UAE obtained a fair score for 1 and 2 h at 60 °C and a fair score for 1 h at 30 °C. Based on the overall score, the UAE at 45 °C for 2 h was selected as the best condition and it can be suggested for the extraction of bioactive compounds from *N. zanardini*.

#### 3.3.3. Microwave-Assisted Extraction (MAE)

This section presents a data-driven fuzzy system that ranks different MAE conditions for *N. zanardini* according to the same variables. As presented in Table 4, the experts recommended the MAE at 300 W for 15 min for DPPH and TFC; MAE at 300 W for 10 min for TPC, TPhC, and time; and MAE at 300 W for 20 min for extraction yield as the best extraction conditions. Based on TPC, TPhC, and time, the MAE at 300 W for 10 min scored “good” to “very good”. This condition also achieved a “fair” score (>50) for DPPH, extraction yield, and power parameters and had a “poor” score in TFC. MAE at 300 W for 15 min had good to very good scores for DPPH, TPC, TPhC, and time. It showed “fair” performance regarding extraction yield and power, and “poor” performance in TFC.

Table 7 indicates that MAE at 300 W for 15 min had a total score of good (73), and it can be used to extract bioactive compounds from *N. zanardini*, depending on the intended use. As a result of this method, the perfect score is also displayed.

#### 3.3.4. Ranking of Extraction Methods Based on Fuzzy Logic

To select the most effective extraction method, several factors must be considered. Some of these factors are the plant matrix properties, time, yield, temperature, sample composition, power, and cost [55,56]. According to the previous sections, different extraction methods were recommended based on the parameters. As observed in the previous sections, the most suitable extraction method depends on each parameter. As a result, deciding on the appropriate extraction method is complicated [53,54]. The current study utilized a data-based fuzzy system to rank different extraction techniques from *N. zanardini* according to their antioxidant activity (DPPH), TPC, TPhC, TFC, extraction time, extraction yield, temperature, power, and cost. The fuzzy ranking method provides a flexible approach to ensuring consistent quality in the extraction of bioactive compounds for functional food ingredients. By accounting for uncertainty and variability, it enables manufacturers to optimize extraction processes, improving yield, bioactivity, and process consistency, which is crucial for maintaining the quality and health benefits of bioactive-rich foods and supplements [29].

The expert panel’s responses are shown in Table 5. Based on this table, the experts recommended MAE for DPPH, TPC, TPhC, EY, and time, and ME for TFC, temperature, and cost. Generally, MAE performed better than “good” based on DPPH, TPC, TPhC, time, and cost. Furthermore, this method achieved a “fair” score (>50) for the TFC parameter. According to the ME method, TPC, TPhC, temperature, and cost parameters were considered “good” and somewhat better than “fair” for TFC and EY. UAE had fair to good results on DPPH, TPC, TPhC, and temperature; it had “very poor” yield, TFC, and time results. This method was also “very good” regarding cost.

According to Table 5, different extraction methods were proposed based on several parameters as the most appropriate. As presented in Table 5, based on DPPH, TPC, TPhC, temperature, time, and cost, the MAE scored “good” to “very good”. This method also achieved a “fair” score (>50) for TFC and extraction yield. ME for TFC and temperature had a “fair” performance and, for cost, had a “good” to “very good” score. UAE had fair to good scores for DPPH, TPC, TPhC, and temperature. It showed “fair” to “poor” performance regarding extraction yield, TFC, and time, and “good” performance in cost.

The importance of various parameters must be determined to rank the methods [53]. The importance of each parameter is shown in Table 6. Based on the results of the defuzzification, all extraction parameters were rated as important to very important (defuzzification score >75). Thus, the chosen parameters were appropriate and relevant factors for ranking the different extraction systems. According to the triple scores, cost and temperature are the most important parameters, and extraction yield is the least important parameter (triple scores: (68 93 100); (62 87 99); (51 76 96); defuzzified values: 90, 84.83, and 75.17, respectively). High temperatures have a detrimental effect on bioactive compounds, including phenolic compounds and phlorotannin compounds, and hence on the antioxidant activity of extracts. It should be noted that, despite extraction yield, TPC, and TFC having lower scores than the other parameters, they were nevertheless considered important parameters (score > 75).

**Table 6:** Input parameters importance scores using fuzzy logic methods.

Sensory Attribute	V.UI	UI	SI	I	HI	Scores Triplet	Defuzzification
AA	0	0	0	14	11	(61 86 100)	84.17
TPC	0	0	0	18	7	(57 82 100)	80.83
TPhC	0	0	2	17	6	(54 79 98)	78
TFC	0	0	4	16	5	(53 78 97)	77
EY	0	0	3	17	5	(51 76 96)	75.17
Time	0	0	2	13	10	(58 83 98)	81.33
Power	0	0	1	15	9	(58 83 99)	81.5
Temperature	0	0	1	11	13	(62 87 99)	84.83
Cost	0	0	0	7	18	(68 93 100)	90

V.UI: very un-important; UI: un-important; SI: somewhat important; I: important; HI: highly important.

**Table 7:** Different extraction method scores of each treatment (the first stage, fuzzy).

Method	Treatments	Scores Triplet	Defuzzification	Rank
ME	60 °C 12 h	(22.86 46.43 70.86)	46.71	V
	60 °C 24 h	(25 48.43 73.14)	48.86	II
	45 °C 12 h	(21.71 44.71 69.71)	45.38	VI
	45 °C 24 h	(25.43 47.43 71.57)	48.14	IV
	30 °C 12 h	(24.86 48.14 72.86)	48.62	III
	30 °C 24 h	(29.29 52.43 76.29)	52.67	I
UAE	1 h 30 °C	(28.29 51.14 75.29)	51.57	III
	2 h 30 °C	(23 42.43 66.57)	44	VI
	1 h 45 °C	(27.29 48 71.57)	48.95	V
	2 h 45 °C	(29.86 54.43 78.29)	54.31	I
	1 h 60 °C	(29.14 53.71 78.71)	53.86	II
	2 h 60 °C	(26.86 50.71 75.43)	51	IV
MAE	200 w 10 min	(34.29 58.14 78.71)	57.59	V
	200 w 15 min	(43.29 68.29 86)	67.07	III
	200 w 20 min	(40.71 65.71 86)	64.93	IV
	300 w 10 min	(44.29 69.29 85)	67.74	II
	300 w 15 min	(49.71 74.71 90)	73.09	I
	300 w 20 min	(36.29 57.14 75.86)	56.79	VI

Based on the combination of Table 5 and Table 6, the overall fuzzy score of each extraction method is shown in Table 8. The ME method showed good efficiency at ambient temperature, resulting in a fair total score for this method. Furthermore, the UAE extract had the lowest TFC and EY. The parameters studied in this study indicate that the UAE does not provide any unique advantage over the ME method. According to our results, MAE has a higher total score (74.89) and can be suggested for extracting bioactive compounds from *N. zanardini* according to the final process (score above 70).

MAE leverages rapid heating, enhanced mass transfer, and selective extraction. These principles lead to improved efficiency, reduced solvent usage, and higher-quality bioactive compound extracts [57]. The MAE method effectively increases yield while preserving the bioactive properties of compounds, such as antioxidants and polyphenols, which are vital for enhancing the nutritional profile, functionality, and shelf life of food products [39].

Fuzzy logic has shown potential in ranking extraction methods. One study used fuzzy logic to evaluate solvents and techniques (e.g., maceration, ultrasound-assisted, ohmic-assisted, and decoction) on the yield, pH, phenolic content, and antioxidant activity of flixweed seed powder, finding ohmic-assisted extraction with water as the most efficient [11]. Another study applied fuzzy logic to rank seedless barberry fruit extraction systems, with cold press, high-pressure CO₂, and a hot water bath scoring above average [10].

## 4. Conclusions

Brown algae, including *N. zanardini*, are rich in biologically active compounds with significant potential for food applications. This study demonstrates that extraction methods strongly influence the extraction yield (EY), antioxidant activity (DPPH), total flavonoid content (TFC), total phenolic content (TPC), and total phlorotannin content (TPhC) of *N. zanardini*. By applying a fuzzy logic system, extraction methods were ranked based on factors like temperature, time, power, and cost, allowing for a flexible and data-driven approach to optimize extraction parameters. This framework ensures precise control, uniformity, and quality in the extraction of bioactive compounds, which is crucial for food applications.

Among the methods evaluated, MAE outperformed ME and UAE, achieving optimal scores across critical factors such as time, cost, and extraction efficiency, with fuzzy logic recommending MAE for high DPPH (56.03%), TPC (895.70 ± 7.54 mg GAE/g), TPhC (943.04 ± 8.48 mg PG/g), and EY (46.54% ± 1.39) in a short time (15 min). Plant-derived antioxidants, including flavonoids, phenols, and phlorotannins, show promise for natural food preservation and extending shelf life, aligning with consumer demand for sustainable ingredients. The integration of MAE with fuzzy logic optimization enhances bioactive compound extraction efficiency, providing a cost-effective and sustainable solution for large-scale food production.

This study highlights the antioxidant potential of *N. zanardini* extracts. Further research should investigate the phytochemical profile, seasonal variability, and broader biological potential of these extracts. Additionally, future work should focus on optimizing food-grade extraction processes that address scalability, cost-efficiency, and product quality during scale-up, to facilitate the translation of laboratory findings into industrial food production.

## Figures and Tables

**Table 1:** Total phenolic content (TPC), total phlorotannin content (TPhC), total flavonoid content (TFC), and extraction yield (EY) of *N. zanardini* extracts.

Extraction Method	Condition	TPC (mg GAE/g)	TFC (mg QE/g)	TPhC (mg PGE/g)	EY (%)
**ME**	30 °C 12 h	391.96 ± 1.85 ^c^	115.19 ± 0.44 ^d^	376.33 ± 2.08 ^c^	25.54 ± 0.73 ^b^
	45 °C 12 h	333.26 ± 0.61 ^d^	129.71 ± 2.72 ^c^	310.29 ± 0.69 ^d^	23.48 ± 0.55 ^c^
	60 °C 12 h	443 ± 1.44 ^b^	94.29 ± 2.20 ^e^	433.75 ± 1.62 ^b^	27.67 ± 0.58 ^a^
	30 °C 24 h	469.15 ± 0.84 ^a^	140.95 ± 0.22 ^b^	463.17 ± 0.95 ^a^	25.45 ± 0.53 ^b^
	45 °C 24 h	307.96 ± 0.67 ^e^	242.67 ± 0.30 ^a^	281.83 ± 0.75 ^e^	26.73 ± 0.38 ^ab^
	60 °C 24 h	391.04 ± 1.61 ^c^	117.38 ± 0.33 ^d^	375.29 ± 1.81 ^c^	26.02 ± 0.94 ^ab^
**UAE**	30 °C 60 min	451.26 ± 5.50 ^b^	71.76 ± 0.22 ^d^	443.04 ± 6.19 ^b^	17.76 ± 0.67 ^c^
	45 °C 60 min	485.96 ± 8.40 ^a^	28.43 ± 0.43 ^f^	482.08 ± 9.45 ^a^	23.35 ± 1.40 ^b^
	60 °C 60 min	341.18 ± 1.23 ^d^	180.33 ± 0.97 ^b^	319.21 ± 1.38 ^d^	23.00 ± 0.91 ^b^
	30 °C 120 min	450.60 ± 2.53 ^b^	32.57 ± 0.14 ^e^	442.29 ± 2.84 ^b^	24.68 ± 1.53 ^b^
	45 °C 120 min	437.93 ± 1.28 ^c^	103.29 ± 2.40 ^c^	428.04 ± 1.44 ^c^	22.34 ± 0.61 ^b^
	60 °C 120 min	347.18 ± 0.23 ^d^	205.29 ± 0.43 ^a^	325.96 ± 0.26 ^d^	28.27 ± 0.81 ^a^
**MAE**	10 min 200 w	411.85 ± 0.36 ^d^	81.43 ± 0.28 ^d^	398.71 ± 0.40 ^d^	33.19 ± 0.76 ^e^
	15 min 200 w	786.93 ± 0.13 ^b^	107.19 ± 1.72 ^c^	820.67 ± 0.14 ^b^	47.23 ± 0.54 ^cd^
	20 min 200 w	410.26 ± 2.75 ^d^	119.57 ± 3.55 ^b^	396.92 ± 3.10 ^d^	51.03 ± 0.46 ^b^
	10 min 300 w	897.22 ± 1.66 ^a^	95.95 ± 0.30 ^c^	944.75 ± 1.87 ^a^	46.15 ± 0.96 ^d^
	15 min 300 w	895.70 ± 7.54 ^a^	134.29 ± 9.26 ^a^	943.04 ± 8.48 ^a^	46.54 ± 1.39 ^c^
	20 min 300 w	713.89 ± 22.64 ^e^	57.90 ± 0.22 ^e^	738.50 ± 25.46 ^e^	54.04 ± 0.84 ^a^

ME: Maceration Extraction, UAE: Ultrasound-Assisted Extraction, MAE: Microwave-Assisted Extraction, TPC: total phenolic content, TPhC: total phlorotannin content, TFC: total flavonoid content. Different symbols indicate statistically significant differences in compound recovery for each method.

**Table 2:** Responses of the expert group for different maceration treatments.

Treatment	V.P (0 0 25)	P (0 25 50)	F (25 50 75)	G (50 75 100)	V.G (75 100 100)	Sensory Scores Triplet	Defuzzification
DPPH (%)							
60 °C 12 h	4	8	13	0	0	(13 34 59)	35.33
60 °C 24 h	1	3	18	3	0	(24 48 73)	48.33
45 °C 12 h	7	10	8	0	0	(8 26 51)	28.33
45 °C 24 h	11	10	4	0	0	(4 18 43)	21.67
30 °C 12 h	6	9	10	0	0	(10 29 54)	31
30 °C 24 h	3	3	17	2	0	(21 43 68)	44
TPC							
60 °C 12 h	0	0	11	12	2	(41 66 89)	65.67
60 °C 24 h	0	0	12	12	1	(39 64 88)	63.83
45 °C 12 h	0	1	16	8	0	(32 57 82)	57
45 °C 24 h	0	2	15	8	0	(31 56 81)	56
30 °C 12 h	0	0	11	13	1	(40 65 89)	64.83
30 °C 24 h	0	0	8	13	4	(46 71 92)	70.33
TPhC							
60 °C 12 h	0	0	11	12	2	(41 66 89)	65.67
60 °C 24 h	0	0	12	12	1	(39 64 88)	63.83
45 °C 12 h	0	1	16	8	0	(32 57 82)	57
45 °C 24 h	0	2	15	8	0	(31 56 81)	56
30 °C 12 h	0	0	11	13	1	(40 65 89)	64.83
30 °C 24 h	0	0	8	13	4	(46 71 92)	70.33
TFC							
60 °C 12 h	1	17	7	0	0	(7 31 56)	31.33
60 °C 24 h	0	10	13	2	0	(17 42 67)	42
45 °C 12 h	0	5	18	2	0	(22 47 72)	47
45 °C 24 h	0	0	4	15	6	(52 77 96)	76
30 °C 12 h	0	10	13	2	0	(17 42 67)	42
30 °C 24 h	0	0	21	4	0	(29 54 79)	54
EY							
60 °C 12 h	1	4	20	0	0	(20 44 69)	44.33
60 °C 24 h	2	2	21	0	0	(21 44 69)	44.67
45 °C 12 h	3	7	15	0	0	(15 37 62)	38
45 °C 24 h	2	3	20	0	0	(20 43 68)	43.67
30 °C 12 h	2	4	19	0	0	(19 42 67)	42.67
30 °C 24 h	2	5	18	0	0	(18 41 66)	41.67
Time							
60 °C 12 h	4	14	7	0	0	(7 28 53)	29.33
45 °C 12 h	4	14	7	0	0	(7 28 53)	29.33
30 °C 12 h	4	14	7	0	0	(7 28 53)	29.33
60 °C 24 h	8	13	4	0	0	(4 21 46)	23.67
45 °C 24 h	8	13	4	0	0	(4 21 46)	23.67
30 °C 24 h	8	13	4	0	0	(4 21 46)	23.67
Temperature							
60 °C 12 h	0	1	17	7	0	(31 56 81)	56
60 °C 24 h	0	1	17	7	0	(31 56 81)	56
45 °C 12 h	0	0	14	11	0	(36 61 86)	61
45 °C 24 h	0	0	14	11	0	(36 61 86)	61
30 °C 12 h	0	0	9	16	0	(41 66 91)	66
30 °C 24 h	0	0	9	16	0	(41 66 91)	66

V.P: very poor; P: poor; F: fair; G: good; V.G: very good.

**Table 3:** Responses of the expert group for different ultrasound treatments.

Treatment	V.P	P	F	G	V.G	Sensory Scores Triplet	Defuzzification
	(0 0 25)	(0 25 50)	(25 50 75)	(50 75 100)	(75 100 100)		
DPPH (%)							
1 h 30 °C	0	0	17	8	0	(33 58 83)	58
2 h 30 °C	15	8	2	0	0	(2 12 37)	17
1 h 45 °C	4	6	13	2	0	(17 38 63)	39.33
2 h 45 °C	0	0	11	10	4	(43 68 89)	67.33
1 h 60 °C	0	0	17	8	0	(33 58 83)	58
2 h 60 °C	7	8	10	0	0	(10 28 53)	30.33
TPC							
1 h 30 °C	0	0	11	11	3	(42 67 89)	66.5
2 h 30 °C	0	0	11	11	3	(42 67 89)	66.5
1 h 45 °C	0	0	7	13	5	(48 73 93)	72.17
2 h 45 °C	0	0	11	12	2	(41 66 89)	65.67
1 h 60 °C	0	0	17	8	0	(33 58 83)	58
2 h 60 °C	0	0	16	9	0	(34 59 84)	59
TPhC							
1 h 30 °C	0	0	11	11	3	(42 67 89)	66.5
2 h 30 °C	0	0	11	11	3	(42 67 89)	66.5
1 h 45 °C	0	0	7	13	5	(48 73 93)	72.17
2 h 45 °C	0	0	11	12	2	(41 66 89)	65.67
1 h 60 °C	0	0	17	8	0	(33 58 83)	58
2 h 60 °C	0	0	16	9	0	(34 59 84)	59
TFC							
1 h 30 °C	8	14	3	0	0	(3 20 45)	22.67
2 h 30 °C	21	4	0	0	0	(0 4 29)	11
1 h 45 °C	23	2	0	0	0	(0 2 27)	9.67
2 h 45 °C	0	11	13	1	0	(15 40 65)	40
1 h 60 °C	0	0	18	7	0	(32 57 82)	57
2 h 60 °C	0	0	13	10	2	(39 64 87)	63.67
EY							
1 h 30 °C	7	9	9	0	0	(9 27 52)	29.33
2 h 30 °C	3	7	15	0	0	(15 37 62)	38
1 h 45 °C	3	8	14	0	0	(14 36 61)	37
2 h 45 °C	3	8	14	0	0	(14 36 61)	37
1 h 60 °C	3	8	14	0	0	(14 36 61)	37
2 h 60 °C	1	3	21	0	0	(21 45 70)	45.33
Time							
1 h 30 °C	0	5	12	8	0	(28 53 78)	53
1 h 45 °C	0	5	12	8	0	(28 53 78)	53
1 h 60 °C	0	5	12	8	0	(28 53 78)	53
2 h 30 °C	0	9	13	3	0	(19 44 69)	44
2 h 45 °C	0	9	13	3	0	(19 44 69)	44
2 h 60 °C	0	9	13	3	0	(19 44 69)	44
Temperature							
1 h 60 °C	0	1	17	7	0	(31 56 81)	56
2 h 60 °C	0	1	17	7	0	(31 56 81)	56
1 h 45 °C	0	0	14	11	0	(36 61 86)	61
2 h 45 °C	0	0	14	11	0	(36 61 86)	61
1 h 30 °C	0	0	9	16	0	(41 66 91)	66
2 h 30 °C	0	0	9	16	0	(41 66 91)	66

V.P: very poor; P: poor; F: fair; G: good; V.G: very good.

**Table 4:** Responses of the expert group to different microwave treatments.

Treatment	V.P (0 0 25)	P (0 25 50)	F (25 50 75)	G (50 75 100)	V.G (75 100 100)	Sensory Scores Triplet	Defuzzification
DPPH (%)							
200 w 10 min	5	7	12	1	0	(14 34 59)	35.67
200 w 15 min	0	2	20	3	0	(26 51 76)	51
200 w 20 min	0	0	8	12	5	(47 72 92)	71.17
300 w 10 min	0	0	18	7	0	(32 57 82)	57
300 w 15 min	0	0	0	18	7	(57 82 100)	80.83
300 w 20 min	18	7	0	0	0	(0 7 32)	13
TPC							
200 w 10 min	0	0	11	12	2	(41 66 89)	65.67
200 w 15 min	0	0	0	12	13	(63 88 100)	85.83
200 w 20 min	0	0	12	11	2	(40 65 88)	64.67
300 w 10 min	0	0	0	5	20	(70 95 100)	91.67
300 w 15 min	0	0	0	6	19	(69 94 100)	90.83
300 w 20 min	0	0	3	12	10	(57 82 97)	80.33
TPhC							
200 w 10 min	0	0	11	12	2	(41 66 89)	65.67
200 w 15 min	0	0	0	12	13	(63 88 100)	85.83
200 w 20 min	0	0	12	11	2	(40 65 88)	64.67
300 w 10 min	0	0	0	5	20	(70 95 100)	91.67
300 w 15 min	0	0	0	6	19	(69 94 100)	90.83
300 w 20 min	0	0	3	12	10	(57 82 97)	80.33
TFC							
200 w 10 min	3	17	5	0	0	(5 27 52)	28
200 w 15 min	0	11	13	1	0	(15 40 65)	40
200 w 20 min	0	9	14	2	0	(18 43 68)	43
300 w 10 min	0	17	8	0	0	(8 33 58)	33
300 w 15 min	0	5	17	3	0	(23 48 73)	48
300 w 20 min	11	11	3	0	0	(3 17 42)	20.67
EY							
200 w 10 min	0	2	16	7	0	(30 55 80)	55
200 w 15 min	0	5	11	9	0	(29 54 79)	54
200 w 20 min	0	4	8	11	2	(36 61 84)	60.67
300 w 10 min	0	5	12	8	0	(28 53 78)	53
300 w 15 min	0	5	10	10	0	(30 55 80)	55
300 w 20 min	0	3	8	10	4	(40 65 86)	64.33
Time							
200 w 10 min	0	0	0	0	25	(75 100 100)	95.83
300 w 10 min	0	0	0	0	25	(75 100 100)	95.83
200 w 15 min	0	0	0	2	23	(73 98 100)	94.17
300 w 15 min	0	0	0	2	23	(73 98 100)	94.17
200 w 20 min	0	0	0	5	20	(70 95 100)	91.67
300 w 20 min	0	0	0	5	20	(70 95 100)	91.67
Power							
200 w 10 min	0	2	14	7	2	(34 59 82)	58.67
200 w 15 min	0	2	14	7	2	(34 59 82)	58.67
200 w 20 min	0	2	14	7	2	(34 59 82)	58.67
300 w 10 min	0	7	9	9	0	(27 52 77)	52
300 w 15 min	0	7	9	9	0	(27 52 77)	52
300 w 20 min	0	7	9	9	0	(27 52 77)	52

V.P: very poor; P: poor; F: fair; G: good; V.G: very good.

**Table 5:** Responses of the expert group to the various extraction methods and variables.

Treatment	V.P (0 0 25)	P (0 25 50)	F (25 50 75)	G (50 75 100)	V.G (75 100 100)	Sensory Scores Triplet	Defuzzification
DPPH (%)							
ME	3	3	17	2	0	(21 43 68)	44
UAE	0	0	11	10	4	(43 68 89)	67.33
MAE	0	0	0	18	7	(57 82 100)	80.83
TPC							
ME	0	0	8	13	4	(46 71 92)	70.33
UAE	0	0	11	12	2	(41 66 89)	65.67
MAE	0	0	0	6	19	(69 94 100)	90.83
TPhC							
ME	0	0	8	13	4	(46 71 92)	70.33
UAE	0	0	11	12	2	(41 66 89)	65.67
MAE	0	0	0	6	19	(69 94 100)	90.83
TFC							
ME	0	0	21	4	0	(29 54 79)	54
UAE	0	11	13	1	0	(15 40 65)	40
MAE	0	5	17	3	0	(23 48 73)	48
EY							
ME	2	5	18	0	0	(18 41 66)	41.67
UAE	3	8	14	0	0	(14 36 61)	37
MAE	0	5	10	10	0	(30 55 80)	55
Time							
ME	8	13	4	0	0	(4 21 46)	23.67
UAE	0	9	13	3	0	(19 44 69)	44
MAE	0	0	0	2	23	(73 98 100)	94.17
Temperature							
ME	0	0	9	16	0	(41 66 91)	66
UAE	0	0	14	11	0	(36 61 86)	61
MAE	0	7	9	9	0	(27 52 77)	52
Cost							
ME	0	0	0	8	17	(67 92 100)	89.17
UAE	0	0	0	10	15	(65 90 100)	87.5
MAE	0	0	0	10	15	(65 90 100)	87.5

V.P: very poor; P: poor; F: fair; G: good; V.G: very good. ME: Maceration Extraction, UAE: Ultrasound-Assisted Extraction, MAE: Microwave-Assisted Extraction.

**Table 8:** Different extraction method scores of *N. zanardini* (the second stage, fuzzy).

Treatments	Scores Triplet	Defuzzification	Rank	Top Field
ME	(34 57.37 79.25)	57.12	III	TFC, Temp, and Cost
UAE	(34.25 58.87 81)	58.45	II	-
MAE	(51.62 76.62 91.25)	74.89	I	DPPH, TPC, TPhC, EY, and Time

## Data Availability

The data presented in this study are available on request from the corresponding author (accurately indicate status).
